# Forecasting next season’s *Ixodes ricinus* nymphal density: the example of southern Germany 2018

**DOI:** 10.1007/s10493-018-0267-6

**Published:** 2018-05-30

**Authors:** Katharina Brugger, Melanie Walter, Lidia Chitimia-Dobler, Gerhard Dobler, Franz Rubel

**Affiliations:** 1 Institute for Veterinary Public Health, University of Veterinary Medicine Vienna, Veterinärplatz 1, 1210 Vienna, Austria; 2 Bundeswehr Institute of Microbiology, Neuherbergstraße 11, 80937 Munich, Germany; 3 German Center of Infection Research (DZIF), Partner Site Munich, Munich, Germany; 4 Parasitology Unit, University of Hohenheim, Emil-Wolff-Straße 34, 70593 Stuttgart, Germany

**Keywords:** Tick-borne diseases, Tick-borne encephalitis, Lyme borreliosis, Mast seeding, Fructification

## Abstract

The castor bean tick, *Ixodes ricinus* (L.) (Ixodida: Ixodidae), is the principal vector of pathogens causing tick-borne encephalitis or Lyme borreliosis in Europe. It is therefore of general interest to make an estimate of the density of *I. ricinus* for the whole year at the beginning of the tick season. There are two necessary conditions for making a successful prediction: a long homogeneous time series of observed tick density and a clear biological relationship between environmental predictors and tick density. A 9-year time series covering the period 2009–2017 of nymphal *I. ricinus* flagged at monthly intervals in southern Germany has been used. With the hypothesis that *I. ricinus* density is triggered by the fructification of the European beech 2 years before, the mean annual temperature of the previous year, and the current mean winter temperature (December–February), a forecast of the annual nymphal tick density has been made. Therefore, a Poisson regression model was generated resulting in an explained variance of 93.4% and an error of $\hbox {RMSE} = 21$ ticks per $100\,\hbox {m}^2$ (annual $\hbox {MEAN} = 260$ collected ticks/$100\,\hbox {m}^2$). An independent verification of the forecast for the year 2017 resulted in 187 predicted versus 180 observed nymphs per $100\,\hbox {m}^2$. For the year 2018 a relatively high number of 443 questing *I. ricinus* nymphs per $100\,\hbox {m}^2$ is forecasted, i.e., a “good” tick year.

## Introduction

The question whether or not the upcoming year will be a tick year in Europe, i.e., a year with many *Ixodes ricinus* (L.) (Ixodida: Ixodidae) ticks, comes up every spring. If reliable such a forecast would be of great interest for any nature lover, but also for dog owners. This also rises the question whether from such an annual assessment directly the risk level of getting a tick-borne disease such as tick-borne encephalitis (TBE) or Lyme borreliosis (LB) can be derived regardless of other influencing factors.

With four live stages (egg, larva, nymph, and adult) and a life span of up to several years (Gray et al. 2016), a prediction on how the biological system *I. ricinus* will behave over the next few days, months or even years is rather complex. Each time scale has its own challenges and its own accuracy, as already known from meteorology forecasts with different forecast ranges (World Meteorological Organization 2010). The weather situation and all the relevant parameters can be described in detail for the next 2 h (nowcasting) or for the next 12–72 h (short-range forecasting). Otherwise the weather situation can be described less detailed for a period beyond 3 and up to 10 days (medium-range forecast). For periods from 30 days up to 2 years (long-range forecast) averages or deviations are given.

Applied for ticks, short-range forecasts of tick activity cover up to 1 week ahead and should be regularly updated. Influenced mainly by the actual weather, an estimate of tick activity including probable significant changes has to be given. Until now different methods were presented to forecast tick activity in European countries. Temperature and relative humidity turned out to be the main predictors for all methods. While the TICKPRO program releases a single estimate the daily tick activity for the Czech Republic (Daniel et al. 2010), the FleaTickRisk (Beugnet et al. 2009) as well as the TEKENRADAR model (Garcia-Martí et al. 2017) provide a spatial estimate of the tick activity weekly for Europe and daily for The Netherlands, respectively. Only the previous weekly forecasts for Germany provided by the company TICK-RADAR also considered the recent host-seeking tick activity by observing ticks in field plots (Kahl and Dautel, personal communication). Short-range forecasts of tick activity can be used to derive also the risk of acquiring a tick-borne infection for exposed people.

On the other hand, long-range forecasts of tick density covering the coming months or years are still missing. Note that, for these longer time periods short-time or inter-daily fluctuations are not considered. Therefore, long-range forecasts can be interpreted as a guide for decision-making over a longer period. One important requirement for developing long-range forecasts, i.e. predictions of the annual tick density, is an existing monitoring of monthly tick activity over long periods. Although in Germany time series of regularly flagged nymphal ticks at 69 sites are available (Brugger et al. 2016), the majority covers only 2–3 years. So far, the time series of the site Haselmühl (Bavaria, Germany) is the longest with nine consecutive years without data gaps. Time series of comparable lengths such as those from Daniel et al. (2015) or Takken et al. (2017) are seldom or not yet published (Kahl and Dautel, personal communication). The Haselmühl time series was already used to demonstrate that the seasonal inter-annual fluctuations of tick activity are most affected by time-lagged and temporal averaged variables, such as temperature and precipitation, rather than by contemporaneous variables (Brugger et al. 2017a). Here we use the available time series to quantify the influence of biotic and abiotic variables on the annual nymphal *I. ricinus* density and to forecast the next year’s density. Beside time-lagged meteorological variables, also the fructification of European beeches (*Fagus sylvatica*), i.e. the annual seed production, was used. The boosting effect 2 years after a mast seeding on the nymphal density is a long-known concept, e.g for *I. scapularis* in the USA (Ostfeld et al. 1996).

The aim of this work is to determine whether a reliable forecast of the tick density for a given location at the beginning of the tick season is possible. Which biotic and abiotic parameters can be used to forecast the next year’s nymphal tick density? Using the example of the sampling site Haselmühl, a first guess to predict the next year’s annual nymphal *I. ricinus* density is undertaken.

## Materials and methods

### Time series of nymphal tick densities and environmental variables

For the analysis we used the data set of the site Haselmühl, a time series of monthly nymphal ticks per $100\,\hbox {m}^2$ for the period 2009–2017. The site as well as the flagging procedure has been described in detail by Brugger et al. (2017a). Here the main focus is on the *I. ricinus* nymphs as this stage plays an important role in the epidemiology of human infections (Gray 1998). Note that, the annual nymphal density is the total number of *I. ricinus* nymphs monthly collected per $100\,\hbox {m}^2$ during 1 year.

Climate variables were taken from the nearest weather station Regensburg-Oberhub (WMO No. 107760) of the German Weather Service (2018). For the period 2007–2017, time series of annual mean values as well seasonal means (i.e. Dec–Jan–Feb, Mar–April–May, June–July–Aug, Sep–Oct–Nov) for the variables temperature and precipitation were aggregated out of daily measurements.

The density of ticks depends also on the availability of suitable hosts. Preferred hosts of *I. ricinus* larvae and nymphs are among others small rodents (Mihalca and Sándor 2013). As no perennial observations of small rodents are available for the study site Haselmühl, the fructification index of the European beech (*Fagus sylvatica*) was applied for indicating the rodent density. Beechnuts are a basic food source for small rodents resulting in population peaks 1 year after mast seeding (Ostfeld et al. 1996; Clement et al. 2009). Given the importance of forestry in Bavaria, this annual index is available back to 1954 (Kronnert et al. 2016). Fructification is defined as the annual seed production and is divided into four classes according to Eichhorn et al. (2017): (1) absent, i.e. no fructification, (2) scarce, i.e. sporadic occurrence of fructification, but not noticeable at first sight, (3) common, i.e. clearly visible fructification, and (4) abundant, i.e. full fructification, also known as mast seeding.

### Statistical analysis

In a first step the correlation coefficients between the annual nymphal *I. ricinus* density and environmental variables were calculated. As some variables are not normally distributed, the Spearman’s rank order correlation coefficient was applied. Additionally, time-lags of 1 and 2 years previous were considered to identify the highest correlation. Finally, a Poisson regression model for the annual nymphal tick density was developed, whereas non-significant variables were removed in a stepwise procedure. Accounting the overdispersion of the time series, a quasi-Poisson GLM was used to correct the standard errors (Zuur et al. 2009). The coefficient of determination for generalised linear models $\hbox {R}^2$ and the root mean square error (RMSE) were applied as goodness-of-fit measures.

All analyses were conducted with the open-source statistical computing environment R (R Development Core Team 2018). The package rsq (Zhang 2016) was used for calculating the coefficient of determination $\hbox {R}^2$.

## Results

**Fig. 1:**
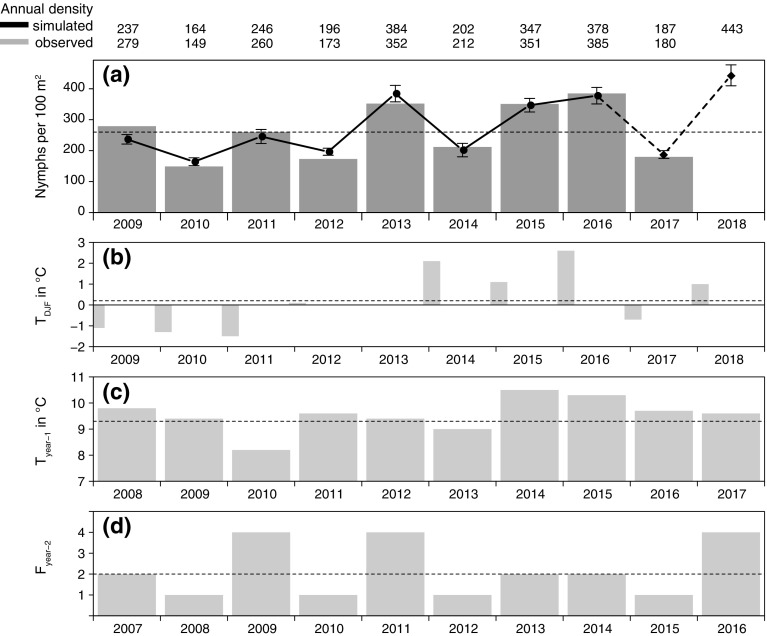
**a** Annual nymphal *Ixodes ricinus* density in Haselmühl (Germany) observed (grey bars) versus simulated (points) between 2009–2017 and forecasted for 2018 (diamond). The associated standard error is given. The explanatory variables **b** mean winter temperature $\hbox {T}_{DJF}$, **c** mean annual temperature of the previous year $\hbox {T}_{year-1}$, and **d** the beech fructification index 2 years prior $\hbox {F}_{year-2}$. The dotted lines indicate the 9-year mean between 2009–2017 for the nymphal tick density and the 30-year mean of 1988–2017 for explanatory variables, respectively

To forecast the nymphal *I. ricinus* density of the next year, a quasi-Poisson model was derived with the mean winter temperature of the months December to February $\hbox {T}_{DJF}$, the mean annual temperature of the previous year $\hbox {T}_{year-1}$, as well as the beech fructification index 2 years prior $\hbox {F}_{year-2}$ as indicated in Table 1. The model results in an explained variance of 93.4% and an RMSE of 21 ticks per year. The latter is equivalent to 8% of the mean collected annual tick density of 260 nymphs/$100\,\hbox {m}^2$. As depicted in Fig. 1, the model simulated the annual nymphal tick density with peaks almost every second year fairly accurately. After a year with relative low tick density below 200 nymphs per $100\,\hbox {m}^2$ in 2017 an annual density of 443 nymphs per $100\,\hbox {m}^2$ was estimated for 2018 (Fig. 1). This forecast can be evaluated by independent data, when observations will be available at the end of the year 2018.

**Table 1:** Summary of the quasi-Poisson regression model for annual tick density

	Estimate	SE	z	p
Intercept	2.5404	0.7031	3.613	$< 0.05$
$\hbox {T}_{DJF}$	0.0911	0.0315	2.893	$< 0.05$
$\hbox {T}_{year-1}$	0.2591	0.0706	3.672	$< 0.05$
$\hbox {F}_{year-2}$	0.2440	0.0351	6.959	$<0.001$

For the explanatory variables mean winter temperature ($\hbox {T}_{DJF}$), mean annual temperature of the previous year ($\hbox {T}_{year-1}$), and the beech fructification index 2 years prior ($\hbox {F}_{year-2}$) the parameter estimates, the standard errors SE, the z-values (test statistics), and the p-values (significance) are given

In a pilot study, the same model was already used to forecast the annual nymphal tick density for the year 2017. As presented at the ${ 19}^{th }$ *Annual Meeting of the International Scientific Working Group on Tick-Borne Encephalitis* (Kunze and ISW-TBE 2017), a relatively low tick density of 187 ticks per $100\,\hbox {m}^2$ was predicted for Haselmühl in March 2017 (Brugger et al. 2017b) and evaluated with 180 collected ticks per $100\,\hbox {m}^2$ at the end of 2017. So far, this can be considered as the most robust model evaluation based on model-independent observations. A limitation of the model, however, is its statistical nature implying that it was fitted to the specific location of Haselmühl (Bavaria, Germany). Therefore, it is not generally applicable and needs individual adjustment when applied to other sites.

## Discussion

**Fig. 2:**
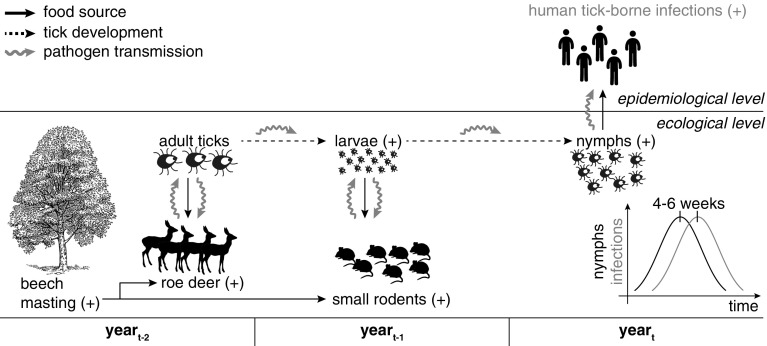
The effect of mast seeding on *Ixodes ricinus* and the possible transmission of tick-borne pathogens. An increase is indicated by (+)

Using the long-term monitoring of *I. ricinus* in Haselmühl, it was demonstrated that the fructification index as well as time-lagged temperature means are appropriate predictors for the annual nymphal tick density. Even more, by the end of the winter time, i.e. at the end of February, a forecast of the tick density for the upcoming year can be made. Especially, mast seeding, i.e. full fructification, turned out to be an appropriate predictor for the annual nymphal tick density. Mast seeding of beeches sets off a chain of ecological reactions (Kelly 1994; Ostfeld et al. 1996). The effects of seeding from the perspective of ticks and tick-borne pathogens is outlined in Fig. 2. As an optimal food source, a magnitude of beech nuts attracts large mammals as roe deer (*Capreolus capreolus*) or wild boar (*Sus scrofa*), which are common hosts for female *I. ricinus* (Gray et al. 2016). As a consequence, this boosts the density of larvae in the following summer. Coincident, the abundant beech nuts benefit an increased survival and breeding of small rodent populations mainly *Myodes* spp. or *Apodemus* spp., which are suitable hosts for *I. ricinus* larvae. Together with a moderate annual temperature in the preceding year and warmer winter temperatures this culminates after moulting from larvae to nymphs and successful overwintering in higher nymphal densities 2 years after mast seeding. Such population peaks can result in an increased risk for humans to acquire tick-borne pathogens causing e.g. TBE or LB. The relation between mast seeding, the subsequent increase of mouse populations and disease incidence one or two years later has been already shown e.g. for human Lyme disease in the Eastern USA (Jones et al. 1998; Schauber et al. 2005) and Poland (Bogdziewicz and Szymkowiak 2016), or Puumala virus infections in Germany (Reil et al. 2015).

As an alternative to the fructification index, the small rodent density or hunting statistics can be applied directly as biotic predictors. For Germany, several time series are available e.g. for field voles (*Microtus agrestis*) or bank voles (*Myodes glareolus*) some of which go back to 1952 (Imholt et al. 2017). However, these data sets were not used in the current study, as the observation periods available for the voles did not match those available for *I. ricinus*. Furthermore, the fructification index is optimal as it is synchronised over both large areas and climate zones (Vacchiano et al. 2017).

A reliable forecasting of the annual tick density can also provide a first estimate of the risk for acquiring a tick-borne pathogen and the related human incidences. In Germany, the large majority of human TBE cases was reported each year in the southern federal states, especially in Bavaria (Robert Koch Institute 2018). A total of 1,361 TBE cases were registered in Bavaria between 2009–2017. The correlation between the annual tick densities in Haselmühl and the TBE cases in Bavaria is relatively high for the period 2009–2016 ($\hbox {r}_S = 0.73$, $\hbox {p} < 0.05$, $\hbox {n} = 8$). However, with the increase of TBE cases in 2017 the correlation drops notably ($\hbox {r}_S = 0.46$, $\hbox {p} = 0.21$, $\hbox {n} = 9$). This unexpected increase of TBE cases in 2017 not only on district level but also on federal state level is inexplicable. One possible explanation is the result of the culmination of annual cycles of different length coinciding (Zeman 2017). Nevertheless, the extraordinarly high number of TBE cases reported in 2017 shows the limitations of correlative models. If a so far unidentified factor or circumstance is not considered, a reliable TBE forecast is not possible yet. One missing predictor might be hunting statistics, as already demonstrated with TBE forecasts based on statistical models for Sweden (Haemig et al. 2011) or Germany (Kiffner et al. 2010). On the other hand also the “good” mushroom season in 2017 could have an effect on the TBE incidences (Jaenson et al. 2012).

Generally, such long-range forecasts of *I. ricinus* densities are essential for epidemiological considerations: not only for individuals at risk of acquiring a pathogen capable of causing TBE or LB, but also for public health authorities.
